# Optimizing survival of *Leptocybe invasa* (Hymenoptera: Eulophidae) parasitoids: effects of nutrition and temperature

**DOI:** 10.1093/jisesa/ieag069

**Published:** 2026-07-03

**Authors:** Thais Alves da Mota, Amanda Rodrigues de Souza, David Lopes Teixeira, José Raimundo de Souza Passos, Barbara de Oliveira Puretz, José Cola Zanuncio, Carlos Frederico Wilcken

**Affiliations:** Laboratório de Controle Biológico de Pragas Florestais (FCA/UNESP), Campus de Botucatu, Botucatu, Brazil; Empresa Gerdau Aços Longos S/A—Gerdau Florestal, Três Marias, Brazil; Departamento de Entomologia/BIOAGRO, Universidade Federal de Viçosa, Viçosa, Brazil; Departamento de Bioestatística, Biologia Vegetal, Parasitologia e Zoologia—Instituto de Biociências (UNESP), Campus de Botucatu, Botucatu, Brazil; Laboratório de Controle Biológico de Pragas Florestais (FCA/UNESP), Campus de Botucatu, Botucatu, Brazil; Departamento de Entomologia/BIOAGRO, Universidade Federal de Viçosa, Viçosa, Brazil; Laboratório de Controle Biológico de Pragas Florestais (FCA/UNESP), Campus de Botucatu, Botucatu, Brazil

**Keywords:** biological control, ectoparasitoid, forest pest management, mass rearing

## Abstract

The distribution and economic impact of the invasive gall wasp *Leptocybe invasa* Fisher & La Salle, 2004 (Hymenoptera: Eulophidae), a major pest of eucalyptus, are global. *Quadrastichus mendeli* Kim & La Salle, 2008 and *Selitrichodes neseri* Kelly & La Salle, 2012 (Hymenoptera: Eulophidae) are effective biological control agents of this pest, but their use depends on optimizing laboratory rearing protocols. This study assessed the longevity and survival of *Q. mendeli* females fed different diets and maintained at temperatures from 18 to 30 °C, and of *Q. mendeli* females and *S. neseri* males and females fed different diets at 24 °C. The experiment was conducted in a completely randomized factorial design. Longevity data were analyzed using generalized linear models, and survival was analyzed using Kaplan–Meier survival curves. Longevity and survival varied according to diet and temperature for *Q. mendeli* females and according to diet at 24 °C for *S. neseri* males and females. *Quadrastichus mendeli* females reaching a maximum longevity of 104.7 d with honey diets at moderate temperature. At 24 °C, *S. neseri* females and males reached maximum longevities of 23.25 d, and 17.00 d, respectively, with honey diets. Longevity declining under starvation or the pollen-only diet. Female longevity exceeded that of males, and *Q. mendeli* lived longer than *S. neseri*. Parasitoid longevity and survival were highest with honey diets at moderate temperatures, providing practical conditions for laboratory maintenance, mass rearing, and release of these parasitoids in biological control programs against *L. invasa*.

## Graphical abstract

**Graphical Abstract:**
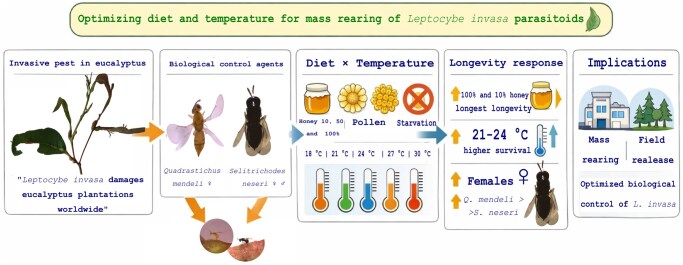

## Introduction

The gall wasp *Leptocybe invasa* Fisher & La Salle, 2004 (Hymenoptera: Eulophidae), an invasive pest of global relevance (Mendel et al. 2004, Nugnes et al. 2015, Ouyang et al. 2024), damages commercial forests of eucalyptus, particularly in Africa, Asia, Oceania, and South America (Zheng et al. 2014, Gevers et al. 2021a, 2021b). Gall formation on the petiole and midrib of leaves and young shoots by *L. invasa* reduces photosynthetic efficiency, suppresses shoot development, and lowers wood production of eucalyptus plants (Roychoudhury 2018, Carvalho et al. 2022, Nag et al. 2025).

The exotic ectoparasitoids *Quadrastichus mendeli* Kim & La Salle, 2008 and *Selitrichodes neseri* Kelly & La Salle, 2012 (Hymenoptera: Eulophidae) (Kim et al. 2008, Kelly et al. 2012, Sangtongpraow and Charernsom 2019) parasitize and kill immature stages of *L. invasa* within galls (Mendel et al. 2017, Huang et al. 2025, Mota et al. 2025), potentially suppressing its populations (Masson et al. 2017, Gevers et al. 2021a, Puretz et al. 2022). In addition, their host-searching efficiency and adaptability to diverse environmental conditions enhance their effectiveness in biological control (Dittrich-Schroder et al. 2014, Masson et al. 2017, Huang et al. 2018).

Field establishment and releases of *Q. mendeli* and *S. neseri* have been reported in eucalyptus-growing regions such as Italy, Brazil, and South Africa. In Italy, these parasitoids have been associated with suppression of *L. invasa* populations (Nugnes et al. 2016), and in Brazil and South Africa they have been recovered from galls or reported after release in biological control programs (Masson et al. 2017, Ndlela et al. 2018, Gevers et al. 2021b). These introductions are mainly associated with classical biological control of this invasive pest. However, reliable laboratory rearing methods are also necessary to maintain colonies, support post-release monitoring, and enable inoculative or augmentative releases when additional field releases are required.

Parasitoid survival is generally higher with diets rich in readily available simple sugars, such as honey, and lower under nutritionally inadequate diets or starvation (Patt and Rohrig 2017, Xiong et al. 2021, Wang et al. 2022). Survival is also inversely related to temperatures below or above the optimal range (Rashki et al. 2020, Moore et al. 2022, Malinski et al. 2023).

Despite the importance of *Q. mendeli* and *S. neseri* as biological control agents of *L. invasa*, information on how adult diet and temperature affect their longevity and survival under laboratory conditions remains limited. This gap restricts the development of standardized protocols for maintaining adults during mass rearing, transport, and prerelease periods. Therefore, this study aimed to evaluate the longevity and survival of *Q. mendeli* females fed different diets and maintained at temperatures from 18 to 30 °C, and of *Q. mendeli* females and *S. neseri* males and females fed different diets at 24 °C. By identifying diet and temperature combinations that improve adult survival, this study provides novel and practical information for optimizing laboratory rearing and biological control programs against *L. invasa*.

## Materials and Methods

### Insect Source and Laboratory Conditions

Eucalyptus branches infested with *L. invasa* were collected in commercial forest plantations in the municipality of São Simão, São Paulo State, Brazil. These branches were placed in wooden cages (79.5 cm high × 44.5 cm wide×39.5 cm deep) arranged on metal shelves in a laboratory maintained at 25 ± 2 °C, 60 ± 10% relative humidity (RH), and a 12:12 h (L:D) photoperiod. Adult emergence of parasitoids was monitored daily, and newly emerged individuals were collected and used in the experiments described below.

### Experiment 1: Longevity and Survival of *Q. mendeli* Fed Different Diets at 5 Temperatures

#### Experimental Design

The experiment followed a completely randomized design (CRD) in a factorial arrangement of 5 × 5, consisting of 5 temperatures and 5 diet treatments. Each treatment combination included 15 replications, with 1 insect per experimental unit.

#### Diet Treatments

Newly emerged *Q. mendeli* adults (<24 h old) were individually placed in flat-bottom glass vials (2.5 cm diameter × 8.5 cm height) sealed with PVC film and provided with one of the following diets: honey solutions at concentrations of 10%, 50%, or 100%; wildflower pollen; or no food. Honey and wildflower pollen used in the experiment were obtained from MAHA Apícola, Botucatu, São Paulo, Brazil. Both products were commercially obtained and contained no added preservatives. The no-food treatment was used as a negative control, hereafter referred to as the starvation treatment, to estimate baseline adult longevity and survival in the absence of external nutritional supplementation. This control was selected because the objective was to compare carbohydrate- and pollen-based diets with complete food deprivation, allowing the direct evaluation of the contribution of each food source to adult survival. Wildflower pollen was included as an alternative solid food source to test whether it could improve adult longevity and survival relative to complete food deprivation, although its suitability for these parasitoids had not been previously established.

Honey solutions were applied directly to the inner wall of the vial using a flat natural-bristle brush (reference 456, no. 0), at approximately 0.5 µL per vial. Wildflower pollen was offered on a moistened piece of filter paper (0.5 × 1.0 cm) inside the vials (Zhang et al. 2004).

#### Environmental Conditions

Vials were maintained in climate-controlled chambers at constant temperatures of 18, 21, 24, 27, or 30 ºC, under a 12:12 h (L:D) photoperiod and 60±10% RH.

#### Longevity Assessment

Insects were observed every 24 h, and mortality was recorded to determine longevity as the number of days from adult emergence until death. Food sources were replaced and vials cleaned every 2 d to prevent contamination and deterioration.

#### Survival Analysis

Survival over time per diet and temperature was evaluated using survival curves, based on daily mortality records.

### Experiment 2: Longevity and Survival of *Q. mendeli* and *S. neseri* Fed Different Diets at 24 °C

#### Experimental Design

This experiment followed a CRD in a factorial arrangement of 3×5 with 3 parasitoid groups (*Q. mendeli* females, *S. neseri* females, and *S. neseri* males) and 5 diet treatments with 15 replications using 1 insect per experimental unit.

#### Diet Treatments and Maintenance

Newly emerged adults (<24 h old) were individually placed in glass vials and submitted to the same diet preparation, food replacement, and container cleaning described in experiment 1: honey solutions at 10%, 50%, or 100%; wildflower pollen; or no food.

#### Environmental Conditions and Longevity Assessment

All insects were maintained at a constant temperature of 24 ºC, under a 12:12 h (L:D) photoperiod and 60 ± 10% RH. Longevity was recorded every 24 h as the number of days from adult emergence until their death.

#### Survival Analysis

Survival curves were constructed per parasitoid group and diet treatment, based on daily mortality data.

#### Statistical Analysis

Longevity data (days) were analyzed using generalized linear models (GLMs) with a gamma distribution and a logarithmic link function (Nelder and Wedderburn 1972). The effects of diet, temperature, and their interaction on the longevity of *Q. mendeli* females were included in the model for experiment 1, and the effects of parasitoid group, diet, and their interaction were evaluated for experiment 2.

Analyses were performed using the GENMOD procedure in SAS University Edition (SAS Institute Inc.). Means of the significant effects detected were compared using the Tukey–Kramer test at a 5% significance level (Westfall et al. 1999).

Survival curves were estimated using the Kaplan–Meier method (Lee et al. 2004), and differences between treatments were assessed using the Log-rank test with Sidak adjustment for multiple comparisons (Westfall et al. 1999).

## Results

### Longevity and Survival of *Q. Mendeli* Fed Different Diets at 5 Temperatures

The GLM indicated that the longevity of *Q. mendeli* females was significantly affected by diet (χ^2^ = 608.36, df = 4, *P* < 0.0001), temperature (χ^2^ = 147.67, df = 4, *P* < 0.0001), and the diet × temperature interaction (χ^2^ = 102.88, df = 16, *P* < 0.0001). Longevity of *Q. mendeli* females was greatest with 50% and 100% honey, particularly at 21 ºC, with values of 104.7_** **_± 7.77 and 98.8 ± 6.82 d, respectively, and lowest under starvation or with wildflower pollen, with values of 1.7 ± 0.16 and 6.8 ±_** **_1.23 d, respectively (Table 1).

**Table 1.** Longevity (mean ± SE) of *Quadrastichus mendeli* (Hymenoptera: Eulophidae) females under starvation, wildflower pollen, or honey diets (10%, 50%, 100%) at temperatures (Temp.) from 18 to 30 ºC (RH 60±10%, 12 h photophase)

Temp.	Starvation	Pollen	Honey		
			10%	50%	100%
**18 °C**	4.1 ± 0.45 Ab	6.8 ± 1.23 Ab	39.1 ± 9.16 Aa	37.2 ± 7.09 Ba	28.9 ± 5.24 Ba
**21 °C**	2.8 ± 0.22 ABd	2.8 ± 0.11 Bd	29.6 ± 9.39 Abc	98.8 ± 6.82 Aab	104.7 ± 7.77 Aa
**24 °C**	2.5 ± 0.29 ABc	2.5 ± 0.24 Bc	13.8 ± 4.01 BCb	43.2 ± 8.10 Ba	44.9 ± 7.79 Ba
**27 °C**	2.1 ± 0.23 ABc	2.4 ± 0.27 Bc	12.7 ± 2.91 Cb	24.7 ± 3.09 Bab	33.9 ± 2.45 Ba
**30 °C**	1.7 ± 0.16 Bb	1.7 ± 0.18 Bb	3.9 ± 0.41 Db	28.1 ± 2.07 Ba	26.8 ± 2.38 Ba

Means followed by the same lowercase letter per row or uppercase letter per column do not differ by the Tukey–Kramer test (*P* < 0.05).

**Fig. 1.**
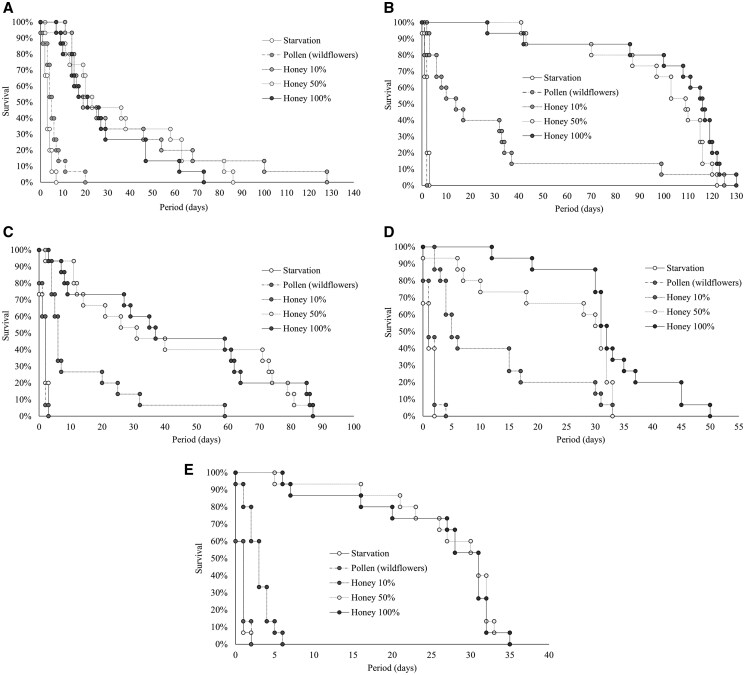
Survival period (days) of *Quadrastichus mendeli* (Hymenoptera: Eulophidae) females at temperatures of 18 ºC A), 21 ºC B), 24 ºC C), 27 ºC D), and 30 ºC E) under starvation, pollen, or honey diets at 10%, 50%, and 100%, under a 12-h photophase and 60±10% relative humidity.

Survival curves differed significantly among diets at all temperatures evaluated: 18 ºC (Log-rank test: χ^2^ = 84.23, df = 4, *P* < 0.0001), 21 ºC (χ^2^ = 87.19, df = 4, *P* < 0.0001), 24 ºC (χ^2^ = 89.86, df = 4, *P* < 0.0001), 27 ºC (χ^2^ = 83.04, df = 4, *P* < 0.0001), and 30 ºC (_**χ**_^2^ = 93.25, df = 4, *P* < 0.0001). Survival of *Q. mendeli* females was higher with honey diets, especially at moderate temperatures, and lower under starvation or with the pollen diet (Fig. 1).

### Longevity and Survival of *Q. mendeli* and *S. neseri* Fed Different Diets at 24 °C

The GLM indicated that longevity differed significantly among parasitoid groups (χ^2^ = 28.47, df = 2, *P* < 0.0001), diets (χ^2^ = 313.29, df = 4, *P* < 0.0001), and the parasitoid group × diet interaction (χ^2^ = 27.25, df = 8, *P* = 0.0006). Female *S. neseri* longevity was greatest with 100% honey (23.25 ± 3.15 d), followed by 50% and 10% honey (12.94 ± 1.68 and 14.44 ± 2.92 d, respectively), and lowest with pollen or starvation (2.44 ± 0.13 and 2.19 ± 0.10 d, respectively) (Table 2).

**Table 2.** Longevity (mean ± SE) of *Quadrastichus mendeli* females and *Selitrichodes neseri* females and males (Hymenoptera: Eulophidae) at 24 ºC under starvation, pollen, or honey diets at different concentrations (RH 60±10%, 12 h photophase)

Diets	*Q. mendeli ♀*	*S. neseri ♀*	*S. neseri ♂*
**Starvation**	2.53 ± 0.29 Ca	2.19 ± 0.10 Ba	1.81 ± 0.13 Ba
**Pollen**	2.47 ± 0.24 Ca	2.44 ± 0.13 Ba	2.37 ± 0.34 Ba
**10% honey**	13.80 ± 4.01 Ba	14.44 ± 2.92 Aa	11.13 ± 1.95 Aa
**50% honey**	43.20 ± 8.10 Aa	12.94 ± 1.68 Ab	17.00 ± 3.48 Ab
**100% honey**	44.93 ± 7.79 Aa	23.25 ± 3.15 Aab	16.00 ± 3.03 Ab

Means followed by the same uppercase letter per column or lowercase letter per row do not differ (*P* < 0.05) by the Tukey–Kramer test.

**Fig. 2.**
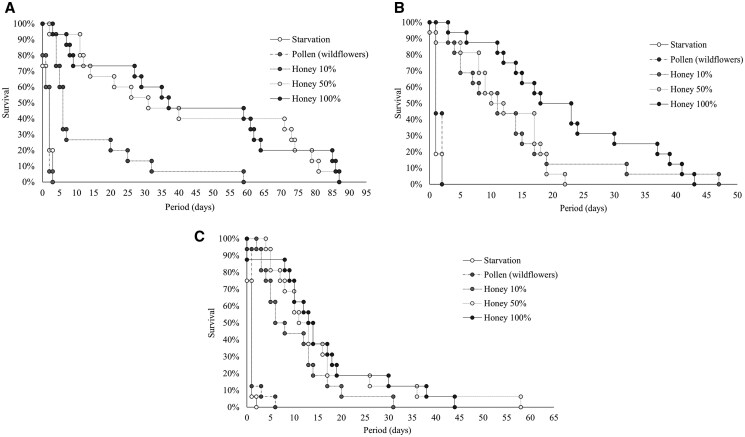
Survival curves (days) of *Quadrastichus mendeli* (Hymenoptera: Eulophidae) females A) and *Selitrichodes neseri* females B) and males C) with different diets: no food, pollen, and honey at 10%, 50%, and 100%, at 24 ºC, 12-h light period, and 60±10% relative humidity.

Longevity of *S. neseri* males was lower than that of females across diets, with the highest value recorded with 50% honey (17.00 ±_** **_3.48 days), followed by 100% honey (16.00 ±_** **_3.03 d) and 10% honey (11.13 ±_** **_1.95 d), and the lowest values with pollen and starvation (2.37 ±_** **_0.34 and 1.81_** **_±_** **_0.13 d, respectively) (Table 2; Fig. 2). Survival curves differed significantly among parasitoid group × diet combinations (Log-rank test: χ^2^ = 274.85, df = 14, *P* < 0.0001), with honey-fed parasitoids surviving longer than those maintained under starvation or with the pollen diet.

## Discussion

Adult longevity and survival are key traits for parasitoid performance because they influence the period available for host location, mating, dispersal, and oviposition (Benelli et al. 2017, Patt and Rohrig, 2017, Irvin and Hoddle 2021). For biological control agents, especially small hymenopteran parasitoids, the availability of suitable adult food sources and adequate thermal conditions can determine whether adults survive long enough to contribute effectively to rearing systems and field-release programs (Eliopoulos et al. 2005, Gómez-Torres et al. 2014, Chen et al. 2022). Therefore, identifying diets and temperatures that extend adult survival is relevant not only for *Q. mendeli* and *S. neseri* but also for the broader development of rearing protocols for parasitoids used in biological control.

### Longevity and Survival of *Q. Mendeli* Fed Different Diets at 5 Temperatures

The greater longevity of *Q. mendeli* females fed 50% and 100% honey, particularly at 21 ºC, indicates more favorable conditions for their energy balance and physiological stability (Zhang et al. 2014, Souza et al. 2018, Sangtongpraow and Charernsom 2019), with higher energy availability and moderate thermal environment maximizing survival (Eliopoulos et al. 2005, Gómez-Torres et al. 2014, Sheng et al. 2019). This is similar to findings for *Closterocerus chamaeleon* Girault, 1922 (Hymenoptera: Eulophidae), a parasitoid of the gall wasp *Ophelimus maskelli* Ashmead, 1900 (Hymenoptera: Eulophidae), with longevity ranging from 36.8 to 127 d when fed 100% honey at the temperatures of 15 ºC and 24 ºC (Garcia et al. 2019, Sinulingga et al. 2021). On the other hand, the reduced longevity of *Q. mendeli* females under starvation or with the pollen diet suggests a dependence on readily available liquid energy sources (Sangtongpraow and Charernsom 2019). Pollen was included as an alternative solid food treatment to determine whether it could improve adult survival relative to complete food deprivation. However, the low survival observed with the pollen diet indicates that pollen alone was unsuitable for adult *Q. mendeli*, probably because these parasitoids depend mainly on readily assimilable liquid carbohydrates and may have difficulty ingesting or metabolically using solid particles (Benelli et al. 2017, Dindo et al. 2019, Hu et al. 2024b). Longevity and biological performance of adult *Tamarixia radiata* Waterston, 1922 (Hymenoptera: Eulophidae) were also higher with diets of simple sugars (Patt and Rohrig 2017, Irvin and Hoddle 2021, Chen et al. 2022).

The higher survival of *Q. mendeli* at 21 ºC than at 30 ºC, even with sugar-rich diets, suggests thermal stress at 30 ºC (Sangtongpraow and Charernsom 2019, Rashki et al. 2020, Xia et al. 2025). Higher temperatures likely increase metabolic rate and accelerate depletion of energy reserves, as proposed for *Aphidius matricariae* Haliday, 1834 (Hymenoptera: Braconidae) (Käfer et al. 2012, Denis et al. 2013, Rashki et al. 2020). The reduction in survival with increasing temperatures, particularly with 10% and 50% honey diets, indicates that sugar intake was insufficient to counteract the deleterious effects of warmer environments (Souza et al. 2016, Moore et al. 2021, Moore et al. 2022). The survival of *Q. mendeli* depends on diet quality and adequate temperature conditions.

### Longevity and Survival of *S. neseri* Fed Different Diets at 24 °C

The greater longevity of *S. neseri* females with 100% honey, followed by 50% and 10% honey diets, and lowest values with pollen or starvation reinforces its reliance on rapidly assimilable energy sources, such as the simple sugars in honey, for metabolic functions, as reported for *Eretmocerus hayati* Zolnerowich & Rose, 2008 (Hymenoptera: Aphelinidae), *Pteromalus puparum* Linnaeus, 1758 (Hymenoptera: Pteromalidae), and *T. radiata* (Zhang et al. 2014, Irvin and Hoddle 2021, Xiong et al. 2024). The greatest longevity with honey concentrations reinforces *S. neseri* dependence on simple sugars, possibly due to limited energy reserves acquired during the larval stage or a higher energetic demand in its adults (Dittrich-Schroder et al. 2014, Masson et al. 2017, Sheng et al. 2019). The inefficacy of pollen may be related to its low digestibility or nutritional inadequacy for parasitoids without developed mouthparts reducing efficient consumption of solid particles (Gallego et al. 2016, Picciau et al. 2019). The longevity of *S. neseri* depends on the intake of concentrated sugar solutions.

The shorter longevity of *S. neseri* males compared with females across diets, especially with 50% and 100% honey, indicates sexual dimorphism, as reported for the solitary parasitoids *Brachymeria lasus* Walker, 1841 (Hymenoptera: Chalcididae) and *Microplitis mediator* Haliday, 1834 (Hymenoptera: Braconidae) (Harvey et al. 2014, Amat et al. 2017, Liu et al. 2019). Sex-related differences may be associated with physiological and behavioral factors, such as greater energy investment by females, increasing their longevity for host searching and oviposition, as reported for *Anastatus disparis* Ruschka, 1921 (Hymenoptera: Eupelmidae) (King et al. 2018, Liu et al. 2019, Lê et al. 2023). The shorter longevity of *S. neseri* males may be due to its more restricted reproductive role concentrated at early adult stage as observed for *Alloxysta* Förster, 1869 (Hymenoptera: Figitidae) and *Habrobracon hebetor* Say, 1836 (Hymenoptera: Braconidae) (Harvey et al. 2019, Huang et al. 2020, El-Ela et al. 2021). The shorter longevity of *S. neseri* males may also reflect differences in metabolism or nutrient assimilation, particularly with diets of lower nutritional value (Farahani et al. 2021, Ye et al. 2022, Hu et al. 2024a).

Although increased longevity and survival can improve the operational use of parasitoids, these variables should not be interpreted as direct measures of fitness or biological control efficacy. Fitness-related responses, such as egg maturation, fecundity, host parasitism, offspring production, and field establishment, were not evaluated in this study. This distinction is important because the relationship between longevity and biological control performance may depend on the ovigeny strategy of each parasitoid. *S. neseri* has been reported as a synovigenic species, with females able to oviposit soon after emergence and continue developing eggs and ovipositing throughout adult life (Dittrich-Schroder et al. 2014). For *Q. mendeli*, previous studies have evaluated egg load and realized fecundity, but the present study did not assess egg maturation or ovigeny status (Sangtongpraow and Charernsom 2019). Therefore, the results presented here identify diet and temperature conditions that improve adult maintenance and survival, while future studies should quantify fecundity, egg load, parasitism rates, offspring production, and field performance to determine how these conditions affect reproductive output and biological control efficacy.

The results have practical implications for mass rearing because adult maintenance is a critical step between parasitoid emergence and field release. Honey-based diets, especially at higher concentrations, can be used to increase adult survival during laboratory handling, colony maintenance, transport, and prerelease storage, as observed for parasitoids maintained on sugar-rich diets (Patt and Rohrig 2017, Irvin and Hoddle 2021, Chen et al. 2022). Maintaining adults at moderate temperatures may also reduce thermal stress and energy depletion, improving the availability of viable parasitoids for biological control programs (Eliopoulos et al. 2005, Gómez-Torres et al. 2014). In contrast, starvation and pollen-only diets were inadequate for maintaining adults, indicating that these conditions should be avoided in rearing protocols. Thus, the combination of concentrated honey diets and moderate temperatures can improve the operational efficiency of parasitoid production by reducing adult mortality before release.

## Conclusion

The longevity and survival of *Q. mendeli* and *S. neseri*, parasitoids of *L. invasa*, were strongly influenced by adult diet and temperature. Honey-based diets, especially at 50% and 100% concentrations, provided the most favorable conditions for adult maintenance, whereas starvation and pollen-only diets resulted in reduced longevity and survival. *Q. mendeli* females survived for more than 100 d when fed honey at 21 ºC, indicating that moderate temperatures combined with concentrated sugar sources can substantially extend adult survival.

Female *S. neseri* lived longer than males across diets, showing that sex-specific differences should be considered when maintaining this species under laboratory conditions. These results provide practical information for improving mass-rearing protocols by reducing adult mortality during colony maintenance, handling, transport, and prerelease periods. The combination of honey-based diets and moderate temperatures can support the production and maintenance of viable adults, contributing to the optimization of biological control programs against *L. invasa*.
